# By the Way

**Published:** 1911-02-25

**Authors:** 


### By the Way.

### KAFFIR REMEDIES FOR TSETSE-FLY DISEASE.

A letter from Dr. Hine, Bishop of Northern Rhodesia, one of those medical men who have abandoned the cure of men's bodies for that of their souls, was published in the *Bulletin of the Sleeping-Sickness Bureau* recently, and serves to recall some of the old-established native remedies for tsetse-fly disease in animals. The Bishop tells how a Rhodesian pioneer of his acquaintance had four dogs bitten in a tsetse-fly belt. They all began to sicken, and three of them were fed on tsetse-flies. Those three recovered; the fourth one, which had not been thus treated, died in six weeks. This practice is a very old one; Livingstone mentions it in 1857; he says that the old chief, then long dead, who discovered it, did not claim any certainty of cure, but simply that a fair percentage of affected stock thus treated would recover, whereas untreated animals all died. Many of the other early writers on South-Eastern Africa also mention this native belief. It seems just possible that some immunity may thus be produced which might prove of service to agriculturists if it could be established on a secure basis. Another similar article of faith among veteran colonists is that both sucklings and their mothers are immune to tsetse-fly infection until weaning takes place. Further, such young animals suckled on a mother actually exposed to the bites of the fly are believed to remain immune afterwards, and are accordingly highly valued. These points seem worthy of attention from some of the research workers in tropical parasitology.

### "THE PASSING OF ABSINTHE."

It is only a few months since the manufacture and sale of absinthe was prohibited by law in Switzerland as the result of a referendum to the people on the subject. It is now evident that ardent reformers are desirous of introducing a similar measure into the laws of France, since shortly after the text of the following proposed law drawn up by M. Vaillant and a number of his colleagues was presented to the Chamber. "That the penalties enforced by the first article of the law regarding the repression of fraud in the sale of goods and of adulteration of foodstuffs and agricultural produce should be held to apply to (1) those who manufacture, expose for sale, or sell the beverage or liqueur known under the name of absinthe, as well as all drinks, called by whatever name, which are produced in imitation of absinthe. The prohibition of the beverage absinthe and its imitations is to extend to these beverages when in a diluted or sweetened state. (2) Those who manufacture, expose for sale, or sell liqueurs, aperients, and aromatic wines obtained by the maceration of aromatic materials in alcohol or brandy, or by the distillation of aromatic plants macerated in alcohol, or by the addition to an alcoholic liquid of essences, extracts, tinctures, or alcoholates, when the composition of these liqueurs has not been approved of by the Academy of Medicine." If the project passes into law, we fancy we can see trouble ahead for the Academy of Medicine!

### A PATHOLOGICAL BANK-NOTE.

An amusing account of the grave physiological defects with which the new French 100-franc note is afflicted was recently given to the Société Médico-historique by Dr. Durante, and reported in the *Gazette des Hôpitaux*. The doctor thinks that "everything nowadays is degenerate, even models, if one is to judge by those who sat to the designer of the note. In the first place, with regard to the child which occupies the left-hand side of the front of the note. Standing firmly on his left foot, it is only with difficulty that his right touches the ground, and this is the case despite the fact that the leg is quite straight and that the pelvis falls markedly towards the right side, being tilted up to the left naturally. One must conclude that the right lower limb is 8 to 10 centimetres, if not more, shorter than the left. Nevertheless no trace of atrophy can be found, and there can therefore be no question of the arrested development being due to infantile paralysis. Any question of coxalgia can be put on one side owing to the straight manner in which the limb lies with reference to the axis of the trunk. Neither can rickets be incriminated, for no cranial deformity is evident. Achondroplasia will not explain the phenomenon, since there is no shortness of the leg or the thigh. One is therefore forced to the conclusion that the painter must have taken as model a child suffering from arrested development, from a congenital partial micromelia, a very rare affection about which we possess little, if any, information." The author next proceeds to criticise the sheep which this child is holding: "While covered as to its head and trunk with white wool, it appears to be completely bald as to its limbs, which are flesh-coloured. It is, therefore, suffering from some very specially localised skin-disease. Near the child a peasant woman, in the erect position, leans on the central frame of the note with her left arm as far as the elbow. Although the fore-arm is not supported, the hand falls down almost to a right angle. This is obviously a case of extensor paralysis. There still remains, however, the workman who occupies the back of the note. Why is his left hand resting on his right thigh, a position which would seem to necessitate a forward rotation of the shoulder, a rotation which, in fact, is wanting? This position puts one in mind of the monoplegtic who, when he wishes to sit down, brings into position with the other hand, or by means of a rotatory trunk movement throws somehow on to his knees, the paralysed member, which often becomes entangled with the thigh of the opposite side. This workman's hand, moreover, with its palm spread out, its first phalanges extended, and its second and third flexed, puts one in mind of a certain cubital clawing, just as the peasant woman calls to memory radial paralysis. Therefore, to sum up the physiological defects which this new note shows us, there is monoplegia or cubital paralysis of the workman, radial paralysis of the peasant woman, an epilating affection of the sheep, and, above all, a marked partial micromelia of the child."

